# Accepting the Challenge: Helping Schools Get Smarter about Supporting Students’ Creative Collaboration and Communication in a Changing World

**DOI:** 10.3390/jintelligence10040080

**Published:** 2022-10-08

**Authors:** Ronald A. Beghetto, Ed Madison

**Affiliations:** 1 Mary Lou Fulton Teachers College, Arizona State University, Tempe, AZ 85281, USA; 2 School of Journalism and Communication, University of Oregon, Eugene, OR 97403, USA

**Keywords:** 21st century learning, creativity, creative collaboration, creative communication, creative confidence, creative curricular experiences, journalistic legacy challenges, journalistic learning

## Abstract

Although the purpose of schools can be (and has been) debated, one common goal that most people agree upon is that schools can and should play a role in preparing young people for the complexities of the future. This goal is somewhat paradoxical in that the future is unknown. So how might schools prepare young people for the unknowable? The prototypical response has been to design learning experiences based on what is already known in the hope that the knowledge, skills, and experiences in school will be durable enough to equip students for navigating the complexities of the problems they will encounter in the future. Consequently, most of what students learn in schools is predetermined. Although we recognize that some of these experiences can be beneficial for helping students in the future, we assert in this concept paper that schools can (and need to) get smarter about the kinds of educational experiences that students engage with if we are to prepare them for addressing the uncertainty of complex problems that they face now and into the future. More specifically, we open this concept paper by briefly discussing the prototypical curricular experience that schools provide young people and how these experiences sometimes fall short in providing students with the opportunities, experience, and confidence necessary to creatively engage with, resolve, and communicate about their experiences addressing complex problems. We then introduce a collaborative creative curricular experience called *Journalistic Legacy Challenges* (JLC). JLCs can support students in learning how to identify, address, document and communicate about complex problems that can make a difference in their communities and in their own and others’ lives. The experiences offered by JLCs differ from prototypical learning experiences because they require young people to identify problems that matter to them, collaborate with skilled others to address those problems, develop their creative confidence, and learn how to use *journalistic learning* to document and communicate about their work to broader audiences.

## 1. Introduction

We live in increasingly challenging and complex times. Scholars have described life in the current epoch as posing new and pressing concerns that present existential threats to life as we know it (Barr et al. 2022; Leahy et al. 2022). Rapid social, ecological, technological, and geopolitical transformations give rise to critically important questions for how we might better prepare ourselves and future generations for addressing current and impending uncertainties. Schools have long been recognized as important socio-cultural institutions with a central goal of preparing young people to develop into productive local and global citizens.

Although the specific purpose of schools can be (and has been) debated (Hannon and Peterson 2021), most people agree that schools can and should play a role in preparing young people for the future. This goal is somewhat paradoxical in that the future is unknown. So how might schools prepare young people for the unknowable? The prototypical response has been to design learning experiences based on what is already known in the hope that the knowledge, skills, and experiences in school will be durable enough to equip students for navigating the complexities of the problems they will encounter in the future. When students question the relevance of curricular content, a common assurance provided by educators is that they should learn what is being taught because “they will need and use it *someday*.”

Equipped with smartphones offering instant access to unlimited information, the “someday” promise can, however, seem ambiguous, intangible, and disconnected from students’ present reality and lived experiences. Consequently, most of what students learn in schools is predetermined and sometimes perceived as irrelevant (at least with respect to students’ lived reality). Although we recognize that the curricular experiences students typically have in schools can be beneficial for students now and into the future, we assert in this concept paper that schools can (and need to) get smarter about the kinds of educational experiences that students engage with if we are to better prepare them for uncertain futures. 

More specifically, we open by briefly discussing the prototypical curricular experience that schools provide young people and how these experiences can fall short in providing students with the opportunities, experience, and confidence necessary to engage with, resolve, and communicate about their experiences addressing complex problems. We then introduce a collaborative creative curricular experience called *Journalistic Legacy Challenges* (JLC). JLCs can support students in learning how to identify, address, document and communicate about complex problems that can make a difference in their own and others’ lives. The experiences offered by JLCs differ from prototypical learning experiences because they require young people to identify complex problems that matter to them (Getzels 1964; Pretz et al. 2003), creatively collaborate with skilled others to address the problems (Hämäläinen and Vähäsantanen 2011; Moran and John-Steiner 2004), develop their creative confidence (Karwowski et al. 2019) and learn how to use journalistic learning to document and communicate about their work to broader audiences.

## 2. Prototypical Curricular Experiences

One way to understand the prototypical curricular experience in schools and classrooms is to recognize that it tends to focus on sameness and certainty (Glăveanu and Beghetto 2016). More specifically, the same group of students typically are required to work through routine problems in the same way, at the same time, and produce the same result. Indeed, much of what students experience in school is predetermined and focused on individual accomplishments. Moreover, the student experience and products of the work tend to be ephemeral and discarded. Other than what students carry in their memories and document in their class notes, the typical documentation and curation of “what students did and learned” tends to be reduced to a numerical score or letter grade in the teachers’ gradebook and on student’s report cards and transcripts. We are not suggesting that this is the fault of teachers or that there are not individual teachers who have broken the prototypical mold by teaching for and with creativity. Rather, we are highlighting systematic and long-lasting features of the typical curricular experience (see Claxton 2008; Sirotnik 1983; Beghetto and Zhao 2022).

Moreover, when it comes to preparing young people for uncertain futures, we see at least three areas where the typical curricular approach seems to fall short in providing opportunities for students to develop their creative confidence, engage in creative collaboration, and learn how to communicate creatively. *Creative confidence* refers to a belief in one’s ability to think or act creatively in and across performance domains (Karwowski et al. 2019). *Creative collaboration* involves students working and problem solving together by actively seeking out alternative perspectives, working with skilled others, and building on each other’s strengths to produce new ideas (Baruah and Paulus 2019; Etelapelto and Lahti 2008; Lubart 2018; Moran and John-Steiner 2004). Additionally, *creative communication* involves learning how to articulate the merit and value of creative work to various audiences (Glăveanu 2013; Plucker 2022). In what follows, we briefly discuss how the prototypical curricular approach can limit opportunities for developing these components of creativity development.

First, with respect to creative confidence, the typical approach in schools is to have students learn how to efficiently and effectively do what is expected and how it is expected (Beghetto 2018a) by working through routine problems (Pólya 1966), rather than develop confidence in identifying their own problems to solve and their own ways of solving them. Routine problems are “pseudo-problems” (Getzels 1964) because they have already been solved. In a school context, routine problems represent learning “exercises” rather than actual problems (Robertson 2017). This is not to say that routine problems lack educational value. Indeed, routine problems play a prominent and important role in teaching and learning because they help students learn how to address existing problems using standard procedures (Lee and Anderson 2013).

The issue with routine problems arises when they become the predominant type of problem students encounter. In order for students to develop their creative confidence necessary for navigating the complexities of the future, they need to also have opportunities to ‘find’ (Runco and Chand 1994) and work through ‘ill-defined’ problems (Pretz et al. 2003). Indeed, as Pólya (1966) has argued, unlike routine problems, ill-defined or non-routine problems “demand some degree of creativity and originality” (p. 127), because these kinds of problems have some level of uncertainty in how they can and should be addressed. Consequently, for students to develop the self-efficacy necessary to engage, persist, and creatively work through ill-defined problems that they face now and into the future, they need opportunities and experiences that require them to identify and tackle ill-defined problems in otherwise structured and supportive learning environments (Bandura 1997; Beghetto 2018b). Indeed, engaging with ill-defined problems require students to seek out new experiences and accept unpredictability, which have been found to be associated with students who have higher levels of creative-self efficacy (Karwowski 2012). Such experiences are also curiosity driven and curiosity is a trait that has been found in prior research to be positively associated with creative self-efficacy in students (see Karwowski 2012; Puente-Diaz and Cavazos-Arroyo 2018).

Next, with respect to creative collaboration (Baruah and Paulus 2019; Lubart 2018; Moran and John-Steiner 2004), much of the curricula in school tends to be focused on individual learning and individual students demonstrating what they know and are capable of doing on their own. Working with others is often forbidden and sometimes even considered cheating (e.g., “do your own work,” “no talking when working on this assignment,” and so on). Conversely, when students have the opportunity to learn how to creatively work together, they can learn how to build on their own and others’ strengths in order to do more than what they might otherwise be able to do individually (Moran and John-Steiner 2004). Even primary school children can learn how to creatively collaborate to support their learning and creativity development. Rojas-Drummond and colleagues (Rojas-Drummond et al. 2008), for instance, report on a study of primary school students in Mexico City who successfully “learned how to collaborate” and “collaborated to learn” and, in turn, were able to collaboratively produce creative writing and multimedia projects.

This is not to say there is no collaboration in school. Indeed, group work is often a common feature of many classrooms throughout young people’s schooling experience. Collaboration in the form of group work, however, often serves as a very narrow representation of collaborative effort. Indeed, “group work” in schools is often not an experience of creative collaboration, but rather students tend to be assigned to groups by the teacher and typically one or two students do most of the work (Chiriac and Granström 2012). This inequitable distribution of who does the work can even occur in situations where students form their own groups, because the goal is often to meet the criteria as quickly as possible. Consequently, students sometimes get upset when working in groups, because they have concerns about equitable distribution of the work and worry about how the group’s efforts (or lack thereof) will impact their individual grade (and, ultimately, their grade point average). Moreover, once groups are formed, students tend not to have an opportunity to disband and join other groups or collaborate with people outside the classroom, which is often required when identifying and working creatively to solve complex problems (Beghetto 2018a; Moran and John-Steiner 2004). In sum, students have limited opportunities to learn how to creatively collaborate in school, particularly when it comes to working together to identify problems to creatively solve that are important to them and that can make a positive impact on others.

Finally, with respect to creative communication, students also tend to have limited opportunities to learn how to effectively document and communicate their creative work to others (Madison 2015; Plucker 2022). If students have opportunities to learn how to communicate their creative work to diverse audiences and receive feedback on that work, then they can develop important creative self-regulation skills necessary for planning, realistic goal setting, managing emotions, and overall improvement of their creative efforts (Ivcevic and Nusbaum 2017). Again, this is not to say that students do not receive helpful feedback from teachers and peers in school. The focus of that feedback, however, tends to be aimed at making sure students are “on track” to meet expectations in expected ways, rather than supporting students’ creative self-regulation and creative communication skills.

Along similar lines, when students have opportunities to present their final work or projects to others (in the form of class presentations), the focus is often on the finished product and not the creative process that resulted in those products including the setbacks, failures, and learning that they experienced throughout the process. Given that experiencing setbacks and failure can play an important role in promoting thinking skills and creativity (Manalo and Kapur 2018; Kapur 2016; von Thienen et al. 2017), it is important that schools provide students and teachers with opportunities to communicate about both their successes and their failures so that young people can learn how to anticipate and grow from setbacks as well as their accomplishments (Beghetto and McBain 2022).

## 3. Creative Curricular Experiences

Creative curricular experiences (CCEs) represent a shift away from prototypical curricular experiences (PCEs), which tend to have a more *transactional* logic, and toward more *transformative* educational experiences. Transactional educational experiences are based on the logic of: “If you do this work, in this way, then you can expect to receive this grade.” Conversely, *transformative* educational experiences focus on doing creative work that can result in positive changes and contributions to others (Beghetto and Glăveanu 2022; Lubart 2018; Sternberg 2021). CCEs, like many creative experiences, include the following features (adapted from Glăveanu and Beghetto 2020):

1. *open-endedness* (i.e., to-be-determined, emergent, and dynamic features),
2. *nonlinearity* (i.e., multiple, and often non-linear, pathways to successful and creative outcomes),
3. *pluri-perspectival* (i.e., acknowledgement of the value and need to be open to difference), and
4. *future orientation* (i.e., exploration of new, alternative, and not yet realized possibilities of what could or should be).

Unlike the transactional focus of PCEs whereby academic learning often serves as a means to its own end, CCEs provide a means for young people to put their existing and developing academic learning to creative work. Importantly, CCEs can be infused in the everyday curriculum and thereby can serve to democratize creative learning experiences for students who have traditionally not had the access or opportunities to participate in creative learning. Indeed, CCEs can be designed to include *all* students rather than be restricted to only a tiny proportion of students identified and selected for gifted and talented programs or who otherwise have the privilege, access, and opportunity to participate in extra-curricular creative learning experiences.

Fortunately, efforts are underway to broaden CCEs for all students (see Beghetto and Zhao 2022; Boss 2017; Zhao 2021). There are even examples of schools and entire school districts that have made important strides in providing students with opportunities to engage in creative learning by tackling authentic community challenges (Boss 2017). Iowa BIG (https://iowabig.org/, accessed on 27 September 2022), for instance, is an initiative that was developed to partner students with local businesses and organizations to engage young people in solving real-world problems. Example projects include everything from efforts aimed at reducing stigmas about mental illness to using hydroponics to grow food (Cedar Rapids Economic Alliance 2019).

If we are serious about supporting students in their development of creative confidence, collaboration, and communication necessary for navigating increasingly complex problems in a rapidly changing world, then we would argue that *all* schools need to get smarter about the kinds of curricular experiences they provide students. We further assert that CCEs should be available to *all* students and not limited to students in specialized, gifted or extra-curricular programs.

In arguing for the inclusion of CCEs in schools and classrooms, we are not suggesting that existing curricular experiences lack merit and should be abandoned. We are also not arguing for “add-on” curricular experiences as we recognize that educators already operate under severe time constraints and may feel that they are already being asked to do more than what they have time to do. Rather, we assert that schools can get smarter about how curricular time is used and the kinds of curricular experiences offered to students. Educators can infuse CCE’s in their curriculum (see Renzulli 2016) by replacing fully predetermined learning experiences with more creative learning experiences (see also Beghetto 2018b for a more in-depth discussion of how this can be accomplished).

In what follows we introduce a particular type of CCE, called *Journalistic Legacy Challenges* (JLCs), that blend opportunities for students to work together identifying, documenting, and creatively solving ill-defined problems in an effort to make a positive and lasting contribution in and beyond their schools and classrooms.

## 4. Introducing Journalistic Legacy Challenges

**Figure 1:**
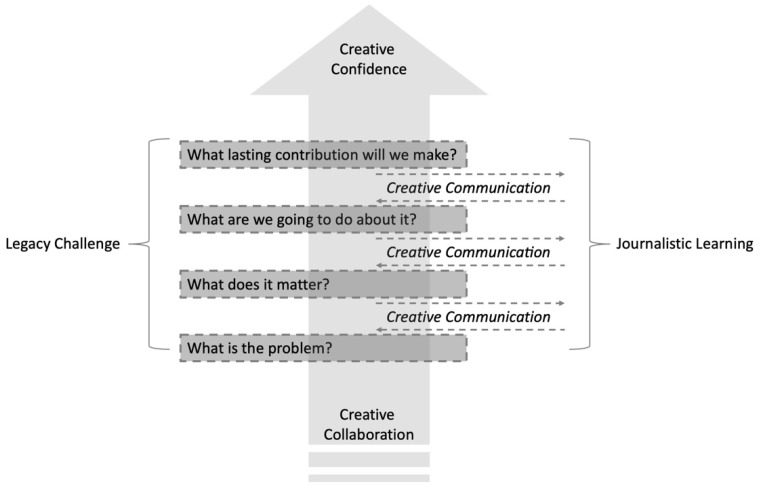
Journalistic Legacy Challenges.

Journalistic legacy challenges (JLCs) represent one-way schools can get smarter about supporting students’ development of their creative confidence, work collaboratively with others, communicate about their creative efforts, and ultimately contribute to others. Figure 1 provides an overview of the various components of JLCs. The two major curricular components of JLCs are the *legacy challenge* framework (Beghetto 2018b) and *journalistic learning* (Madison 2012, 2015). These two major curricular components are driven by creative collaboration, creative communication, and creative confidence. Each of these elements will be discussed in the sections that follow.

## 5. Legacy Challenges

*Legacy challenges* refer to student interest driven projects that provide students with opportunities to put their learning to creative work by allowing them to identify and address complex problems that can make a positive impact in their schools and the world around them (Beghetto 2018b). More specifically, legacy challenges represent a particular type of creative learning experience that provides structured and supportive opportunities for students to:

- *Engage in problem finding—what is the problem?* This feature of a legacy challenge provides students with an opportunity to identify, learn about, and select an ill-defined problem that they and others face in schools, neighborhoods, communities and beyond. As mentioned, these kinds of problems require creativity to identify and solve them (Runco and Chand 1994) because they do not have predetermined solutions or clearly identified procedures for arriving at those solutions (Getzels 1964; Pretz et al. 2003).
- *Creatively communicate about problems—why does the problem matter?* This feature of a legacy challenge framework requires students to develop the skills necessary to creatively communicate (Plucker 2022) to others about the nature of the problem, why it matters, and who it impacts. This requirement differs from prototypical learning experiences because students are expected to develop and articulate their own rationale for addressing a problem rather than being told by someone else why doing the work is important.
- *Creatively collaborate with skilled others—what are we going to do about it?* This third feature of legacy challenges requires students to creatively collaborate with peers, experts, and skilled others to generate possibilities for solving the problem (Moran and John-Steiner 2004; Baruah and Paulus 2019; Etelapelto and Lahti 2008). This aspect of creative collaboration includes identifying and partnering with outside experts and skilled others who can assist students in developing a plan of action to creatively address the problem and monitor their progress along the way. This feature of the legacy challenge also differs from typical curricular experiences because students are collaborating with skilled others to develop their own, creative approach for addressing ill-defined problems.
- *Make a positive and long-term contribution to others—what lasting contribution will we make?* This final feature of legacy challenges requires students to anticipate and evaluate whether the contribution they are making is successful and, most importantly, capable of making a beneficial and long-term impact on others. Unlike typical school-based projects that focus on individual student learning in the short term, legacy challenges require students to actively plan for and monitor the sustainability and positive impact of their work (Beghetto 2018b).

The four features listed above serve as the defining elements of legacy challenges and the last feature refers to the *legacy* component of these kinds of projects because students are expected to take a long-view approach to the work by developing sustainable solutions to problems. A group of graduating high school seniors who develop a food bank for families in their community, for instance, would need to establish a legacy plan to ensure that community partners and younger students (e.g., juniors and sophomores) are involved in the project so that the work carries on after they leave high school. 

One way to think about legacy challenges is that they represent a specific category of creative learning experiences, which can include various existing approaches, such as: design challenges (Brophy et al. 2008; Brown 2009); service learning (Stanton et al. 1999), enrichment activities (Renzulli et al. 2004), playful learning environments (Kangas 2010), and other real-world projects (Boss 2015, 2017). These related approaches can be classified as legacy challenges if they meet the four features of: student identified problems, creative communication about the problems, creative collaboration with skilled others, and making a positive and lasting contribution.

Legacy challenges can range from the work of individual students who identify a social-contextual problem and work with outside partners to solve it to an entire classroom of students addressing a more academic question and contributing their own unique insights. Consider, for instance, Natalie Hampton a teenager in California who, based on her own experience, recognized a problem of social isolation and bullying that often manifests in kids having to sit alone at lunch. In response, Hampton developed an app called “Sit With Us” that provides teenagers who experience bullying and isolation to discreetly find other kids to sit with in the lunchroom (Drake 2016). The legacy of this work is evident in the number of people (100,000+ across seven countries) who have downloaded the app since its launch (TYA 2021). Another example of an impactful legacy project involved a group of students at Blackawton Primary School in Devon, England who collaborated with their teacher and a visiting neuroscientist to publish an article based on their questions about and observations of bee behavior in *Biology Letters*, one of the Royal Society’s top academic journals (Yong 2010). Collaborating with supportive and skilled adults, the students were able to make a positive and lasting scientific contribution. 

## 6. Journalistic Learning

A unique aspect of what we are proposing in this concept paper is that the creative learning of legacy challenges can be further enhanced by infusing aspects of *journalistic learning* (Madison 2012, 2015) into legacy projects. Journalistic learning involves granting students permission to explore topics aligned with their intrinsic interests, thereby fostering authentic student-driven experiences. In turn, students learn how to apply principles and strategies from journalism practice to work with multiple sources of information and communicate their ideas and insights to broader audiences. Part of this learning includes students developing a more active and critical understanding about information and that all sources of information are not equal. More specifically, students learn that claims must be evidence-based and verified. They also learn that one of journalism’s highest aims is to serve the public good. This civic imperative extends beyond simply reporting the day’s news to informing the public to affect positive, societal change.

In the context of journalistic legacy challenges (JLCs), students apply journalistic principles to the ways in which they communicate about the importance of the problems they are attempting to address and in reporting on the work and impact of their projects. In this way, we assert that JLCs can enable students to more effectively and responsibly collaborate and communicate with others using various forms of media. Such efforts align with recommendations of professional educational associations (e.g., National Council of Teachers of English, NCTE 2022), which have called for greater curricular emphasis on media education and the importance of learning how to use information to inform public discourse and societal sense making. This is particularly important given that in the internet age the level of “noise,” misinformation, and even malicious efforts by some to seed discontent requires that young people learn how to be more critical consumers and ethical producers of information (Madison and DeJarnette 2018).

Moreover, with respect to academic accomplishments, there is also correlational evidence that students, particularly students of color, who have had an opportunity to develop and apply principles of journalistic learning in school, tend to also have more successful academic and writing experiences (Dvorak 1990; Dvorak and Choi 2009). Recent research, however, has indicated that journalism classes tend to attract students who are already more academically accomplished (Bobkowski et al. 2017). It is therefore worth exploring in future research whether systematically incorporating aspects of journalistic learning in legacy projects will benefit the academic confidence and competence of more students (beyond those who typically self-select into journalism courses).

Indeed, only a small fraction of students typically have opportunities to learn and apply principles of journalism in school (Madison 2012, 2015). JLCs, however, can provide many more students with opportunities to learn and apply key principles of journalistic learning in and across various subject areas. The problems that students address in JLCs can and often are informed by multiple academic topics, including math, the sciences, history, literature, and the arts. We recognize that educators, particularly those who have little to no experience in journalism, may need to develop their own journalistic skills or partner with colleagues who have the requisite knowledge or even professional journalists to support students’ journalistic learning. Fortunately, educators who are interested in developing their own and their students’ journalistic knowledge can turn to promising self-guided professional development resources.

The *journalistic learning initiative* (JLI), for instance, offers a series of self-guided online professional development modules that can help educators and students develop their capacity to learn and apply journalistic principles (see https://journalisticlearning.com, accessed on 27 September 2022). Since 2015, JLI has been adopted by more than fifty middle and high schools in Oregon, California, Arizona, Idaho, and Washington state. Evaluative data collected from students involved in the program indicates a variety of positive outcomes including, positive perceptions and attitudes, high levels of individual interest, engagement, and persistence in journalistic research and writing, flow experiences in the writing process, high levels of autonomy, competency for critical thinking, and relational support from their teachers and peers as a result of their participation in journalistic learning (Madison et al. 2019).

Journalistic learning, when combined with legacy challenges, blends the unique features of the journalistic approach with students’ engagement in creative problem solving to make a difference in the world around them. Telling the story of one’s creative work is often viewed as a completely subjective and retrospective activity (i.e., people tell their version of the story after they have had the experience). When it comes to JLCs, however, students can learn how to apply professional journalistic standards and skills necessary to document and start telling the story of their creative process from the outset of their work. 

## 7. Creative Communication in JLCs

JLCs can help students more systematically develop their *creative communication* skills by learning how to effectively communicate about and receive feedback on their own unique and meaningful perspectives and ideas. Learning how to be a more effective communicator in the context of JLCs can also help students learn how to document and articulate the creative impact of their work (Plucker 2022). In this way JLCs serve as a vehicle for students to develop their mini-c or subjective creative ideas into creative contributions that benefit others (Kaufman and Beghetto 2009).

As mentioned, JLCs provide opportunities for creative communication from start to finish. Indeed, prior to students embarking on a JLC, students can learn how to communicate about and learn from their own and others concerns about failure and setbacks. Given that JLCs require young people to creatively engage with uncertainty, it can be expected that they will encounter setbacks and failures along the way (von Thienen et al. 2017). One way to anticipate and prepare young people for setbacks and how they might productively respond to failures (Kapur 2016)—even emotionally painful ones—is to share stories of “favorite failures” (Beghetto and McBain 2022). Sharing stories of favorite failures represents a form of creative communication because doing so not only describes examples of failure, but also describes what that failure felt like, and what people who experienced that failure learned from the experience and about themselves. Sharing favorite failures at the outset of JLCs can thereby help young people anticipate potential setbacks, establish strategies for supporting each other when encountering setbacks, and recognize the importance of documenting and creatively communicating their own experiences to others. 

Creative communication in JLCs continues as students work on the identification of the ill-defined problem they want to address. Indeed, students’ need to be able to communicate about the problem they are working on so that other people understand what the problem is and why it is important to address. Students also need to be able to creatively communicate about their projects with outside audiences to develop partnerships, receive helpful feedback, and obtain material support and resources to help them address the problem they identified. Creative communication also plays an important role after students have developed and implemented their creative solutions. When students learn how to document and share the stories of their projects, they can more effectively articulate whether and how their efforts have made a positive impact on others and what they learned from the process.

Taken together JLCs can allow students to creatively communicate their efforts to multiple audiences across time and contexts (Plucker 2022). The creative communication that occurs in JLCs serves as a constant feedback loop—the story students develop and tell others helps external audiences and partners understand the project. As students continually communicate about their stories with others, they put themselves in a position to receive timely feedback that can help them to develop and improve their ideas and efforts, which can support them in the early stages of their work by requiring them to clarify and refine the problems they have identified and plan on addressing.

Constant creative communication can also help students identify new and unique facets of the project that might result in changes in how they think about and act on the problems they have identified. As they continue through the project, constant communication with others can help students reflect on the value and merit of their work and ultimately consider whether and how their creative actions are making a positive and sustainable difference to others, including what aspects of their work they might need to adjust or modify. When students learn how to document and creatively communicate with others about the twists, turns, zigs, zags, pivots and persistence in their projects (Sawyer 2013), the story of their work becomes a creative contribution in itself, because it can contribute new and meaningful insights and understandings to others and even inspire others to engage in their own JLCs.

## 8. Creative Collaboration in JLCs

JLCs also represent *creative collaborations* (Baruah and Paulus 2019; Lubart 2018; Moran and John-Steiner 2004) from start to finish. Given that JLCs represent opportunities to productively engage with ill-defined problems, students will need to partner with people in and beyond the classroom to receive support, feedback, and guidance on their work. When identifying a problem to solve, creative collaboration can be helpful to students in generating possibilities of issues and problems that they might address. Similarly, creative collaborations can help students gain useful and different perspectives on problems they have identified, helping them to clarify why the problem matters, who it is impacting, and why it needs to be addressed.

Creative collaboration is perhaps most clearly needed when students start to consider how they might address the problem they identified. Although young people may be able to identify a complex problem on their own and even develop a compelling rationale, when they move towards solving it they likely will need to partner with people who have more experience, expertise, and resources to actually address the problem. Indeed, having sufficient domain knowledge is critically important when it comes to successful creative problem solving and producing creative contributions (Baer 2020). In this way, creative collaboration helps broaden the possibilities of what kinds of problems young people can address, because even though they may not yet have the experience, domain knowledge and resources necessary to solve an ill-defined problem themselves, they can collaborate with outside experts who can assist them.

## 9. Creative Confidence in JLCs

Finally, JLCs can help young people develop their *creative confidence*, which compliments their development of creative competence. Prior theory and research (see Karwowski and Kaufman 2017) has demonstrated that transforming creative potential into creative action requires creative confidence (and related self-beliefs). Indeed, just because someone can take creative action on a problem, does not mean that they will. Creative action is risky and is often marked with setbacks and, even, emotionally painful failures. The findings of a set of recent studies, for instance, suggests that unless people see the value in doing creative work, have confidence in their creativity, and are willing to take the creative risks necessary to persist in the face of setbacks, then it is unlikely that they will convert their creative potential into creative action (Beghetto et al. 2021; Karwowski and Beghetto 2019).

As discussed, students typically have limited opportunities in school to develop their creative confidence by identifying and creatively solving ill-defined problems. In order for students to develop their creative confidence they therefore need to have opportunities to both observe others (i.e., relatable models, Bandura 1997) and, most importantly, participate themselves in experiences that require creative thought and action. JLCs provide students with structured and supportive opportunities to identify and work through the kinds of problems and issues that require creative solutions and sustained creative action. Moreover, because JLCs provide opportunities for students to creatively engage with uncertainty in an otherwise supportive and structured environment, students have the opportunity to take sensible, creative risks.

When creative risk taking and setbacks are normalized and expected, students likely will be more willing to trust themselves and others (Grant and Coyle 2018) to take on the uncertainties inherent in JLCs. The small successes they have in addressing and working through the challenges can then accrue in the form of creative confidence (see Amabile and Kramer 2011), which in turn can support them in developing their competence in identifying and addressing complex and ill-defined problems they face now and into the future.

## 10. Conclusions

In this concept paper we have asserted that students do not need to wait until they develop domain expertise before they can do meaningful and impactful work. Rather, when schools provide students with creative curricular experiences, such as Journalistic Legacy Challenges, they offer young people opportunities to work with more skilled others and outside experts to put their academic learning to creative use by identifying and addressing complex, real world problems.

As we have discussed, the kinds of experiences offered by JLCs require students to creatively collaborate and communicate with others as well as develop their creative confidence and competence in making a difference in the world around them. We encourage educators and researchers to work together to explore and systematically test-out whether and how the infusion of JLCs can provide students with opportunities to identify complex problems, take creative action, and make an impact on others.

As discussed in the outset of this concept paper, incorporating JLCs in the curriculum does not require completely abandoning existing curricular experiences, rather JLCs can be infused in the everyday curricula by replacing existing assignments and activities with JLC projects. Even spending five minutes a day, over the course of the school year, may provide meaningful opportunities for *all* students to develop their creative confidence, communication, and collaboration skills. Moving forward, researchers and teachers can work together to assess what kinds of opportunities already exist and possible areas that can be opened in the curriculum for JLCs and evaluate progress. Formative evaluation approaches such as those described in Renzulli et al. (2021) can be helpful in supporting these efforts.

In conclusion, we stress that it is not students who need to get smarter before they address complex challenges, rather, it is schools that need to get smarter in the kinds of curricular opportunities they provide young people. If this is the case, then we would argue that schools have a responsibility to provide and support opportunities, like JLCs, for students so that they can learn how to identify and creatively address complex issues and problems they face now and into the future.
